# Peripartum cardiomyopathy among cardiovascular patients referred for echocardiography at Parirenyatwa Teaching Hospital, Harare, Zimbabwe

**DOI:** 10.5830/CVJA-2016-043

**Published:** 2017

**Authors:** Ellise Tapiwa Gmabahaya, James Hakim, Noleen Munyandu, Jonathan Matenga, David Kao

**Affiliations:** College of Health Sciences, University of Zimbabwe, Harare, Zimbabwe; College of Health Sciences, University of Zimbabwe, Harare, Zimbabwe; College of Health Sciences, University of Zimbabwe, Harare, Zimbabwe; College of Health Sciences, University of Zimbabwe, Harare, Zimbabwe; Division of Cardiology, Department of Medicine, University of Colorado Denver School of Medicine, Colorado, USA

**Keywords:** peripartum cardiomyopathy, Zimbabwe, outcomes

## Abstract

**Objectives:**

The main aim was to evaluate the outcome of patients with peripartum cardiomyopathy (PPCM) within six months of diagnosis. The secondary aim was to describe demographic and clinical characteristics of patients with PPCM in Harare, Zimbabwe.

**Methods:**

This was a prospective cohort study in which patients recruited into a detailed PPCM registry were followed up for six months. Echocardiograms were performed at enrolment, and three and six months after diagnosis, to determine left ventricular function.

**Results:**

From 1 August 2012 to 31 July 2013, 43 patients with a new diagnosis of PPCM were recruited at Parirenyatwa Hospital in Harare. At six months, mean ejection fraction improved from 29.7 ± 9.8 to 44.9 ± 14.9%, p < 0.001 and New York Heart Association (NYHA) functional class improved significantly (p < 0.0001). Five (11.6%) patients died.

**Conclusions:**

Left ventricular function improved in a substantial number of patients (42.9%) in this Zimbabwean cohort compared to other African cohorts. However the mortality rate remained high.

## Objectives

Cardiovascular disease has reached epidemic proportions in sub-Saharan Africa and is a major contributor to morbidity and mortality.^1^ These conditions often affect young women disproportionately, particularly during pregnancy, and they may have a worse prognosis compared to other groups of patients.^2^ Peripartum cardiomyopathy (PPCM) is one such condition that affects previously healthy young women during the most productive years of their lives. This has far-reaching consequences for the patient, children and family unit as a whole.

Virchow recognised heart failure in association with pregnancy as early as the 18th century.^3^ However, it was not until the 1930s when Hull and Hafkesbring formally described the syndrome of heart failure following pregnancy, which they called ‘postpartum cardiomyopathy’.^4^ Demakis and Rahmitoola in 1971 formally defined PPCM and gave criteria for its diagnosis, the basis of which remains today.^5^

Since the original description by Demakis, several studies have assessed the clinical profile of patients with PPCM as well as the natural history of the condition. These studies have been done in a variety of settings with the majority emanating from the United States and South Africa. Data from these studies suggest that PPCM has a variable clinical course. Unlike many other forms of cardiomyopathy, patients with PPCM are known to recover fully from the condition. When it occurs, recovery is rapid, usually within the first six months after diagnosis.^5^

Series from the United States and South Africa show that 21 to 78% of patients with PPCM recover left ventricular function (LVEF ≥ 50%) within six months of diagnosis.^6^-^9^ However a proportion of patients never recovers and requires long-term management of chronic heart failure. These women often have relapses of decompensated cardiac failure that may be severe enough to require cardiac transplantation. Factors shown to be associated with recovery of left ventricular function include Caucasian race, higher New York Heart Association (NYHA) functional class, higher ejection fraction and smaller left ventricular dimensions at presentation.^6^,^10^ Typical causes of death in PPCM patients include progressive cardiac failure and sudden cardiac death, presumably due to arrhythmias and thromboembolic events.^11^

PPCM is known to occur more commonly in African women or those of African descent, but despite the potentially devastating consequences of PPCM, there is very little published data about its outcome in African women outside South Africa, and a few isolated historical reports from Nigeria.^12^ A study conducted in Haiti showed an incidence of PPCM of one in 350 live births, which is at least 10 times that of Western nations.^13^ In South Africa the estimated incidence is one in approximately 1 000 live births.^14^

Given these data, it is expected that Zimbabwe would be a setting with a relatively high prevalence of PPCM, the impact of which may be magnified by poorly resourced public hospitals that make the diagnosis and management of these patients suboptimal. Therefore, it was necessary to conduct the current study to look at the outcome of PPCM in Zimbabwe. Clinical characteristics of Zimbabwean PPCM patients were described, and change in left ventricular function, functional status, and overall survival within six months of diagnosis were evaluated.

## Methods

This was a prospective cohort study conducted at Parirenyatwa Hospital, a tertiary-care teaching hospital in Harare, Zimbabwe. The study protocol was approved by the Joint Parirenyatwa Hospital and College of Health Sciences Research ethics committee as well as the Medical Research Council of Zimbabwe. Informed consent was given by all patients.

Consecutive patients seen between 1 August 2012 and 31 July 2013 in the echocardiography clinic at Parirenyatwa Hospital who fulfilled the entry criteria were enrolled into a detailed PPCM registry. Inclusion criteria were: women aged 16 to 49 years; development of symptoms of heart failure one month prior to and up to five months after delivery where no obvious cause could be established; left ventricular systolic dysfunction with ejection fraction (EF) < 45% or fractional shortening (FS) < 30% on transthoracic echocardiograph. Exclusion criteria were: significant organic valvular disease; systolic blood pressure > 160 mmHg and/or diastolic blood pressure > 100 mmHg.

On enrolment, the following was obtained for each case: demographic data, medical and obstetric histories, drug therapy, clinical examination findings and echocardiographic profile. After the initial assessment, these patients were subsequently followed up and managed for six months at the cardiac clinic at Parirenyatwa Hospital.

The two time points that were of interest for this study were three and six months after enrollment. At each time point the NYHA functional class and drug management were assessed. In addition, a thorough clinical examination was carried out and clinical data were recorded. Echocardiography to assess left ventricular function was repeated at the three- and six-month reviews.

Two-dimensional and targeted M-mode echocardiography was performed using a Hitachi EVB 7500 ultrasound scanner. Echocardiograms were carried out with patients in the left lateral decubitus position. Left ventricular ejection fraction (LVEF) was calculated using left ventricular internal systolic (LVDs) and diastolic dimensions (LVDd). These were measured at the level of the mitral valve leaflet tips in the parasternal long-axis view in accordance with the American Society of Echocardiography guidelines.^15^ A rhythm ECG strip was recorded during echocardiography and LVDd was determined in M-mode at the beginning of the Q wave, and LVSd was determined at the end of the T wave. The valves were carefully interrogated in the four standard views to determine morphology.

Echocardiography was performed by cardiologists or senior clinicians at enrolment and at the six-month review. The threemonth studies were performed by the investigator using a mobile Sonosite ultrasound machine and images were recorded and subsequently reviewed by a cardiologist or senior clinician for accuracy of measurements.

Remarkable recovery was defined as an increase in the LVEF > 20% from baseline and complete recovery as LVEF > 50% after six months. The investigator assigned the NYHA functional class for each patient at baseline and at subsequent follow-up visits. Patients were defined as improvers if they were in functional class I or II or had improved by at least one class at the end of the six-month period.

## Statistical analysis

Study data were collected and managed using Research Electronic Data Capture (REDCap), a secure web-based application designed to support data capture for research studies,^16^ hosted at the University of Zimbabwe College of Health Sciences. These data were exported and analysed using the STATA statistical package (version 10.1, College Station, TX). Discrete variables are presented as n (%), and continuous variables are presented as mean ± standard deviation. A paired ANOVA test was used to compare ejection fraction at baseline and after three and six months. Fisher’s exact test was used to compare NYHA functional class at baseline and after three and six months. Significance was defined as a two-tailed p-value < 0.05 unless otherwise specified.

## Results

**Fig. 1.**
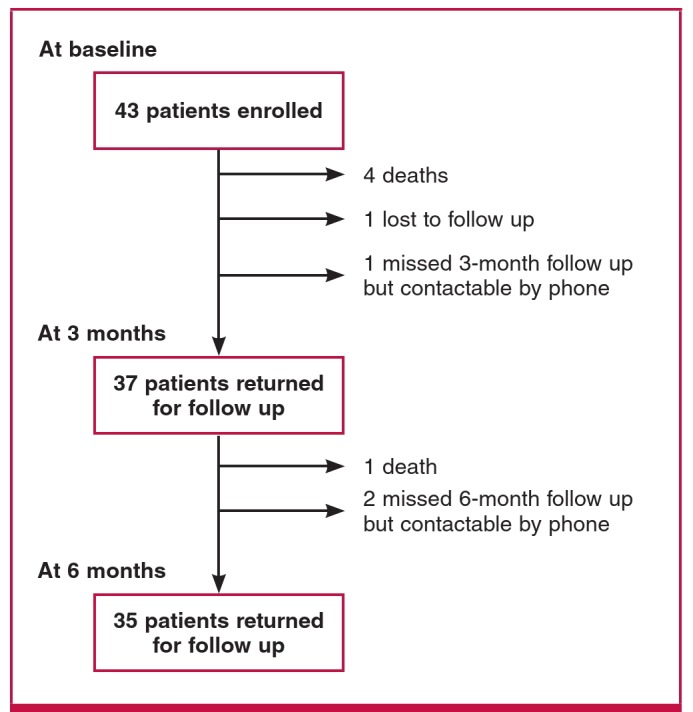
Study flow diagram of 43 participants with newly diagnosed PPCM.

A total of 43 patients were enrolled into the study (Fig. 1). Only one patient was lost to follow up. Left ventricular function at three months could not be assessed for one patient because she did not come for the review, although she had an echocardiogram performed at six months. Two patients missed the six-month review so clinical assessment and echocardiography could not be done. However both patients were contactable by phone and were reported to be alive and well.

Table 1 shows the baseline demographic and clinical characteristics of the patients. The mean age of the cohort was 27.9 ± 6.0 years. The majority of patients (15, 34.9%) were primigravida, with seven (16.3%) having a parity of four or more. At enrolment, 23 (53.5%) of the patients were NYHA class II, with only seven (16.3%) having an NYHA class of IV.

**Table 1:** Baseline demographic and clinical characteristics of study patients

*Variable*	*Frequency n(%)*
Age (years)	27.9 (6)
Parity	
1	15 (34.9)
≥ 2	28 (65.1)
NYHA functional class	
II	23 (53.5)
III	13 (30.2)
IV	7(16.3)
Pregnancy-induced hypertension	15 (34.9)
Gestation type	
Singleton	40 (93.0)
Twins	3 (7.0)
Time of symptom onset	
Pre-partum	1 (2.3)
1–3 months post-partum	40 (9.3)
4–5 months post-partum	2 (4.7)
Echocardiographic data	
Left ventricular end-diastolic diameter (mm, range)	56.8 (43.2–72.2)
Ejection fraction (%, range)	29.7 (4.4–50)*
Left ventricular thrombus	4 (9.3)

*A single patient had an LVEF > 45% but fractional shortening was < 30%.

A relatively large proportion (15, 34.9%) of the cohort had been diagnosed with and managed for pregnancy-induced hypertension. Out of all 43 patients, three had twin deliveries. Only one (2.3%) patient admitted to having symptoms of heart failure two weeks prior to delivery compared to 40 (93.0%) within the first three months of delivery, and two (4.7%) between four and five months postpartum.

On enrolment, all the patients were already on some form of treatment for heart failure; 38 (88.4%) were on diuretics, 28 (65.1%) were on angiotensin converting enzyme inhibitors (ACEIs) or angiotensin receptor blockers (ARBs) and 26 (60.5%) were on spironolactone. In comparison, only eight (18.6%) patients were on beta-blockers (two on atenolol, six on carvedilol). The mean ejection fraction of the cohort at baseline was 29.7 ± 9.8% and four (9.3%) patients had left ventricular thrombi.

Table 2 shows the change in NYHA functional class between the three points. There was a significant improvement in NYHA from baseline to each of the time endpoints (p < 0.001 for both). By three months, 20 (54.1%) patients were in NYHA class I and only two (5.4%) were in NYHA IV. Of the two patients in NYHA IV, one had completely defaulted on treatment and the other had been on suboptimal therapy. By six months, 23 (65.7%) patients had an NYHA class of I compared to only one patient with an NYHA class of IV.

**Table 2:** NYHA class at baseline, and three and six months

**	*Baseline*	*3 months*	*6 months*	**
*Parameters*	*(n = 43), n (%)*	*(n = 37), n (%)*	*(n = 35), n (%)*	*p-value*
NYHA class				< 0.001**
I	0	20 (54.1)	23 (65.7)	
II	23 (53.5)	13 (35.1)	9 (25.7)	
III	13 (20.2)	2 (5.4)	2 (5.7)	
IV	7 (39.5)	2 (5.4)	1 (2.9)	

**Significant at α = 1%

Table 3 summarises the changes in left ventricular function. Patients who completed six months of treatment showed a significant improvement in the ejection fraction from 29.7 ± 9.8% at baseline to 44.9 ± 14.9% after six months (p < 0.001). Increases in LVEF between all time points were statistically significant, except for those that occurred between baseline and three months (p < 0.05).

**Table 3:** Left ventricular systolic function at baseline, and three and six months

**	**	**	**	*0-3 months*	*0-6 months*	*3-6 months*	**
**	*Baseline*	*3 months*	*6 months*	*p-value*	*p-value*	*p-value*	*All p-value*
LVDd (mm)	56.8 ± 6.6	53.9 ± 8.2	53.9 ± 9.2	0.345	0.204	1	0.136
LVEF (%)	29.7 ± 9.8	36.8 ± 13.7	44.9 ± 14.9	0.05	< 0.001**	0.028	< 0.001**

*Significant at α = 5%, **significant at α = 1%.

There was a non-significant reduction in LVDd from 56.8 ± 6.6 mm at baseline to 53.4 ± 9.2 mm after six months. By three months after diagnosis eight (22.3%) of the patients had a normal LVEF and eight (22.3%) showed remarkable LVEF improvement. Of the 35 patients who completed six months of follow up, 15 (42.9%) had normalised left ventricular function. Remarkable improvement of LVEF was seen in 16 (45.7%) patients after six months of follow up.

Of the five (11.6%) patients who died during the study period, four (9.3%) died within the first three months of diagnosis. Two (40.0%) died from progression of heart failure while still hospitalised. Of the three who died outside the hospital, one died of thromboembolic disease, based on a post mortem that showed right leg deep venous thrombosis, a left ventricular thrombus and a large pulmonary embolus. There was no reported cause of death for the other two patients, although one had an intramural thrombus on echocardiography at three months and the other had presumed upper limb deep venous thrombosis, based on clinical examination.

## Discussion

PPCM has never been studied before in Zimbabwe. This study looked at the natural history of this rare condition in a relatively large cohort of Zimbabwean patients with a mean age of 27.9 ± 6.0 years. The majority of the women were primigravidas and a large proportion had been diagnosed with pregnancy-induced hypertension, however none of them were hypertensive at diagnosis.

The LVEF had normalised in a large proportion (42.9%) of the patients and the NYHA functional class had improved significantly after six months of follow up. Still, mortality was relatively high (11.6%), with progressive heart failure and thromboembolic disease being the main causes of death.

Demakis, in his landmark study on PPCM, noted that the condition was ‘more common in the older multiparous woman and in women who have had toxemia and twins’.^5^ Patients who develop PPCM in Zimbabwe have a clinical profile similar to those described in previously published reports, with a few notable exceptions. Firstly, PPCM occurred at a relatively young age in our study. Traditionally, it was generally accepted that PPCM occurred with greater frequency in older compared to younger women. In the United States for example, the mean age of patients who develop PPCM is between 27 and 34 years, with the majority of studies reporting a mean age of more than 30 years.^17^ In the South African cohorts the mean ages ranged from 29 to 31.6 years.^17^ By contrast, the Zimbabwean cohort had a mean age of 27.9 years with the majority of patients (67.4%) being younger than 30 years.

Low socio-economic status and poverty have been consistently linked to younger maternal age.^18^ In Zimbabwe the demographic health survey of 2010 to 2011 showed that the median age for women at marriage was 19.7 years and the median age at first birth was 20.2 years.^19^ This may account for the younger age at presentation of women with PPCM in this cohort.

Secondly, the proportion of patients in their first pregnancy (34.9%) was higher than in most previously published reports. A case in point is the original report by Demakis, which showed that 29% of patients had a parity of either one or two.^5^ In more recent times, 24% of patients from the Haitian and 20% from a South African cohort were primigravidas.^13^,^20^ The reasons for the relatively high proportion of primigravida women in this Zimbabwean cohort is not yet established. It could be due to the relatively large number of young primigravida women inthe Zimbabwean population in general. However, Elkayam et al. also reported a higher proportion of primigravidas (37%) in patients from the USA comprised predominately patients of white or Hispanic ancestry (77%).^21^

Lastly, a relatively large proportion (34.9%) of patients in the current study was diagnosed with pregnancy-induced hypertension (PIH). This figure is much higher than other studies of black patients from Haiti and South Africa, where the proportion of patients with PIH was reported to be 4 and 2%, respectively.^13^,^14^ This is most likely due to the fact that in the three series of patients with PPCM reported by Sliwa and colleagues in South Africa, patients with pre-eclampsia and ‘hypertension of any degree greater than mild’ were excluded from the diagnosis of the condition.^7^,^11^,^20^ This is in contrast to the Zimbabwean cohort in which patients with the whole spectrum of hypertensive disorders of pregnancy were included. However the proportion of patients diagnosed with hypertensive disorders of pregnancy was reported to be higher in the United States.^22^ Women with the whole spectrum of hypertensive disorders of pregnancy were included in the systematic review by Bello et al.^22^ Therefore patients who develop PPCM in Zimbabwe are younger, of lower parity and have a history of gestational hypertension when compared with patients of a similar ethnic background.

Previous studies of PPCM have reported a mixed prognosis for the condition. Data from the United States showed that left ventricular function improved in 35 to 62.2% of patients with PPCM, with most patients recovering within the first six months, although some took up to two years to recover.^17^ The mortality rate in the United States ranged from 1.36 to 18% over variable periods of time.^17^ These studies enrolled mainly Caucasian patients and it was noted that black women had poorer outcomes. In the Haitian study, the rates of recovery were very low with only 24% of women achieving a normal left ventricular function after 2.2 years of follow up. The mortality during the same period of time was 15%.^13^ The result was supported by data from South Africa, which showed that 21 to 23% of patients achieved normal left ventricular function within six months of diagnosis, and between 10 and 27.6% of patients died within six months.^7^,^11^,^20^ In this Zimbabwean cohort, 42.9% of patients had normalised left ventricular function by six months of follow up, with an overall absolute mean change in ejection fraction of 15.2 ± 13.9%. The mortality rate was 11.9%. This is more comparable to figures seen in the Western world where the majority of patients were Caucasian.

There could be several reasons for the better outcome in Zimbabwean patients when compared to patients of similar ethnicity. First, the patients enrolled in the current study were not as sick as patients in the South African and Haitian studies. Only 46.5% of the patients from Zimbabwe had an NYHA functional class of III/IV compared to 69 to 98% of South African and Haitian patients at enrollment. Although not validated, it has been suggested that NYHA functional class could be an independent predictor of left ventricular recovery and is a validated predictor of prognosis.^23^

LVEF at baseline has also been proposed as a predictor of left ventricular recovery and mortality.^6^ However six-month mortality rates in the Zimbabwean cohort were relatively low compared to other South African studies, even though the baseline LVEF of the Zimbabwean cohort (29.7 ± 9.8%) was comparable to that of the South African cohorts, which had baseline ejection fractions of between 25 and 30%.^6^,^7^,^11^,^20^ Mortality rates were also higher in Haitian patients, even though left ventricular function was comparable between Haitian and Zimbabwean PPCM patients (fractional shortening 15 vs 14.3%, respectively).^24^ LVEF also improved by a similar magnitude in all the studies. For example, at the end of six months of follow up, LVEF in the South African patients had increased to between 42.1 and 44.1%,^6^,^7^,^11^,^20^ compared to 44.9% in the Zimbabwean patients. However NYHA functional class at baseline has been shown to more consistently predict mortality in patients with PPCM.^6^,^7^,^25^ The baseline NYHA functional class of patients in the Zimbabwean cohort was lower than that in South African studies.

It has been proposed that patients with hypertensive disorders of pregnancy should be excluded from the definition of PPCM because cardiac dysfunction may be a result of the underlying hypertension rather than a primary cardiomyopathy resulting from the pregnancy.^26^ Hence series of supposed PPCM patients that include women with hypertensive disorders of pregnancy may have a better prognosis and report better outcomes than series that do not. This is because good left ventricular recovery by six months may be less likely to happen in patients with true PPCM.

Although this view may hold true in some cases, it does not make for a strong argument in all cases of PPCM. Other factors such as higher LVEF, smaller left ventricular dimensions at baseline and even Caucasian race may lead to faster recovery of left ventricular function. In addition, this current group of patients was not hypertensive on enrolment, with a mean systolic blood pressure of 117.4 mmHg and mean diastolic blood pressure of 73.1 mmHg. Furthermore, most patients developed symptoms an average of five weeks after delivery, by which time their blood pressures were back to normal.

The degree of recovery of left ventricular function, as measured by the mean LVEF at six months, was similar in South African (42.1–44.1%) and Zimbabwean patients (44.9%) despite the fact that fewer patients in Zimbabwe had access to beta-blockers. Similarly, the proportion of patients who fully recovered left ventricular function in Zimbabwe was similar to Western cohorts. Although beta-blockers have been shown to improve outcomes in patients with systolic dysfunction, their efficacy in patients of African ancestry has been questioned.^27^ A GRK5 polymorphism seen in black patients actually gives genetic beta-blockade and improves survival in African patients.^28^ A meta-analysis also confirmed no significant overall benefit of beta-blockade in black patients with NYHA class III/IV heart failure.^29^

Four of the patients (9%) had intramural thrombi on enrollment, and three out of the five patients who died had thrombotic complications. This is consistent with previous observations that PPCM is a prothrombotic state.^30^,^31^ This supports the recommendation by some experts that anticoagulation should be prescribed to women with PPCM with very low ejection fractions. This is in contrast to recommendations for use of anticoagulation in patients with systolic dysfunction from other causes who are in sinus rhythm.^32^

## Limitations

This study has several limitations. Firstly, the study had a short follow-up period of only six months. In previous studies, the improvement in left ventricular function continued even after the initial six months. Sliwa et al. also showed that the mortality rate actually increased after the first six months, and a proportion of patients who recovered within the first six months still died within two years of diagnosis.^11^ Hence it would have been interesting to observe the long-term outcome in this group of patients.

Secondly, the sample of patients in this study may not have been representative of the patients who develop PPCM in Zimbabwe. For example, only patients who presented for echocardiography were included in this study. Hence women who were not able to get an echocardiogram for various reasons, such as financial constraints, were missed. However it is important to note that Parirenyatwa Hospital was the only public institution offering echocardiography for the whole northern region of the country. The only other public institution offering echocardiography is Mpilo Hospital which is some 400 km from Harare and caters for the southern region of the country. Therefore the catchment area for the study was quite wide although the majority of patients came from in and around Harare.

Lastly, patients presented at different stages in their illness after having received some form of treatment. This was largely due to the fact that most patients presented for investigation only when they had money for the echocardiogram.

## Conclusion

In this study, Zimbabwean PPCM patients were younger and of lower parity than those in previously published studies from Africa, with a relatively high proportion of patients with pregnancy-induced hypertension. The percentage of Zimbabwean patients who recovered left ventricular function by six months was almost double that seen in other PPCM patients with similar ethnicity, although the mortality rate was similar to that observed in other African cohorts. These outcomes occurred despite limited access to medications such as betablockers, which have been shown to improve outcomes in heart failure. A large percentage of patients who died had a high rate of thrombotic complications, supporting the recommendation that patients with PPCM should receive anticoagulation when in the setting of a low ejection fraction. Further research to assess the differences in pathogenesis, treatment and outcomes in Zimbabwean PPCM patients is warranted.
